# Supplementary material for: Characterization of Porcine Ocular Surface Epithelial Microenvironment
Source: Int J Mol Sci. 2023 Apr 19;24(8):7543. doi: 10.3390/ijms24087543 (PMC10145510; doi:10.3390/ijms24087543)
Supplement: Supplementary file 1 [file ijms-24-07543-s001.zip › ijms-2282684-supplementary.pdf]

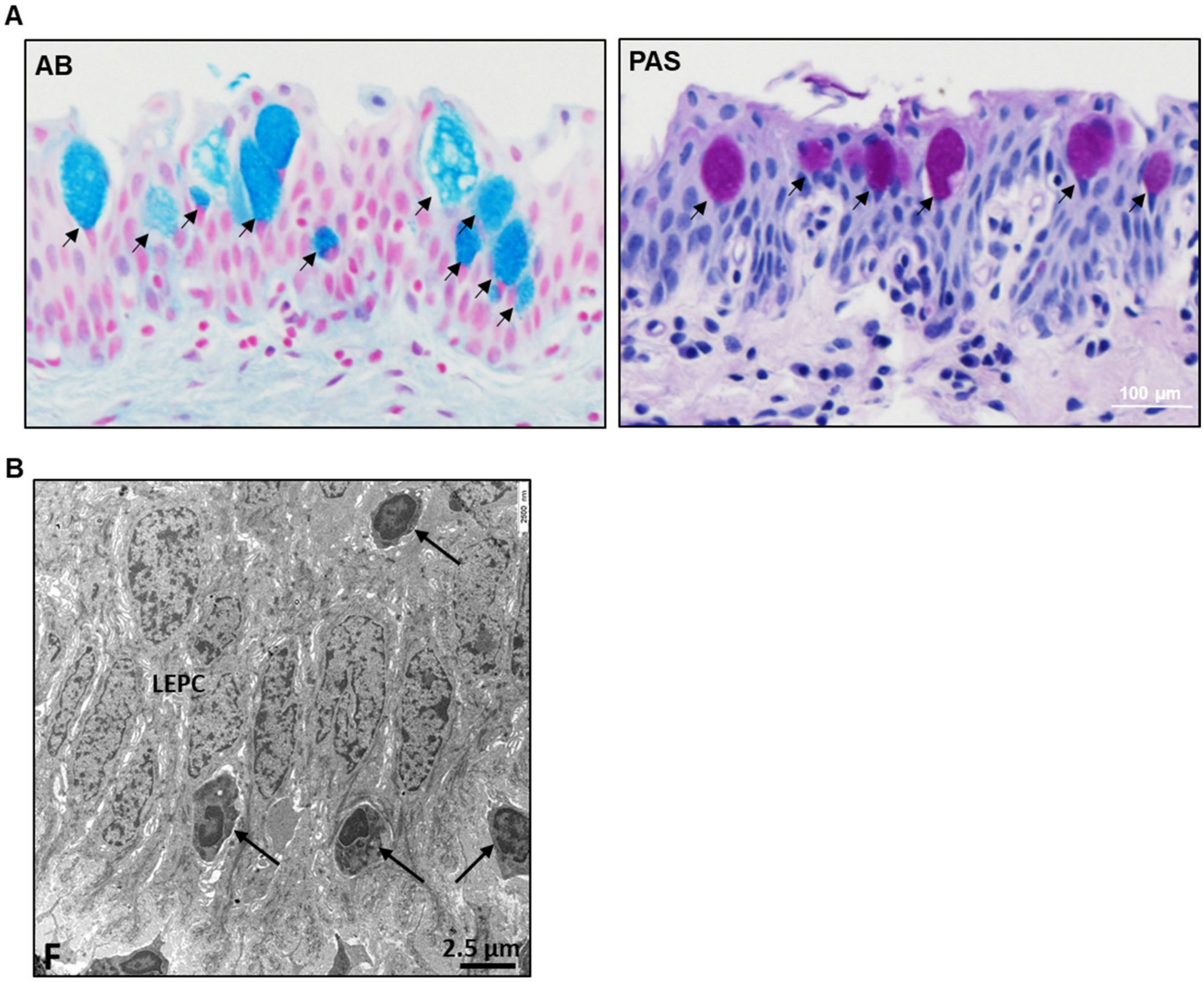

Supplementary Figure S1: (A) Alcian blue and periodic acid-Schiff (PAS) staining of inner eyelid showing the presence of goblet cells (arrows); (B) transmission electron micrograph limbal tissue showing the limbal epithelium intermingled with infiltrating immune cells (arrows).

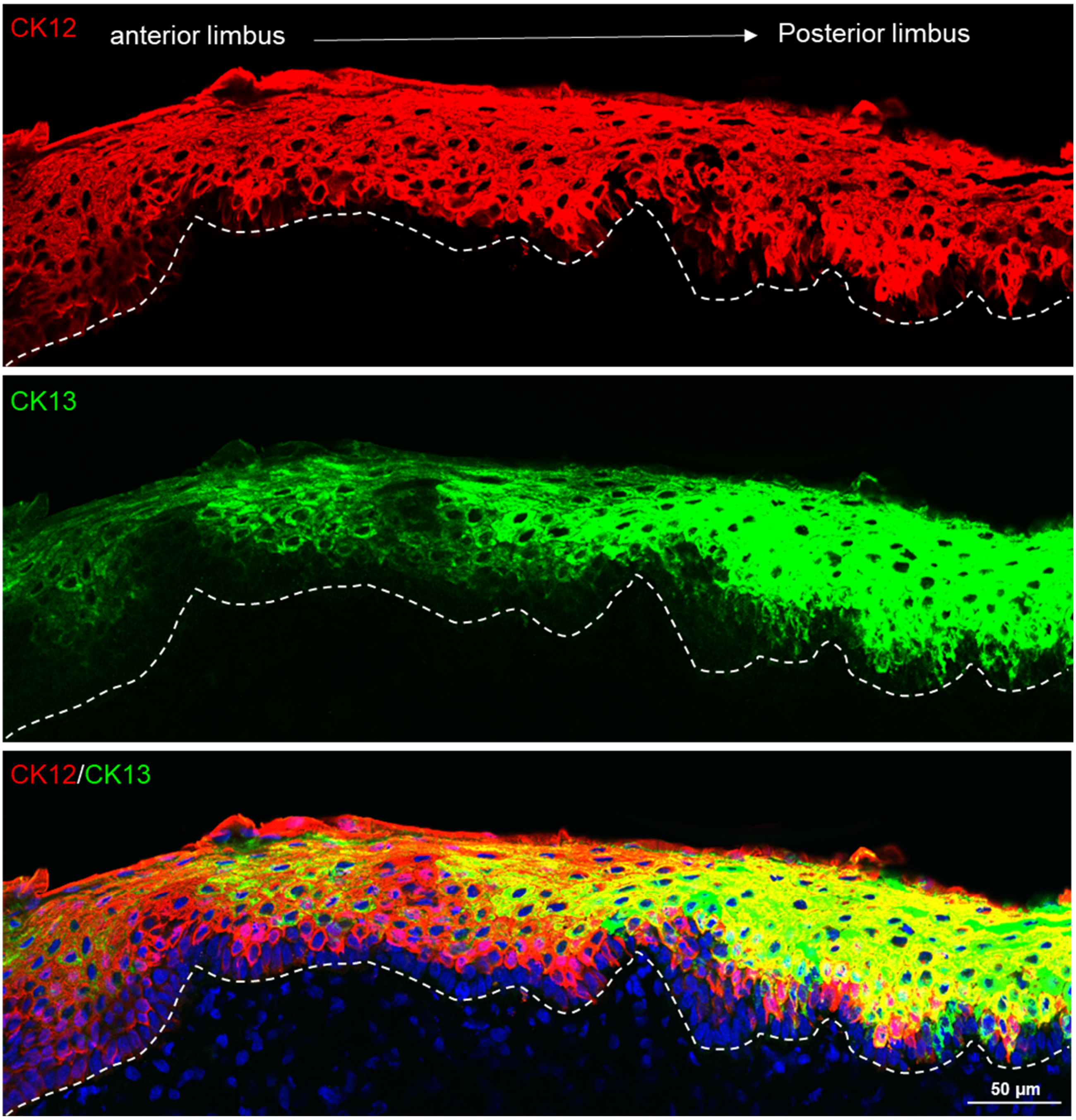

Supplementary Figure S2: Double immunostaining of corneoconjunctival frozen sections showing co-localization of CK12 (red) and CK13 (green) in the suprabasal layers of limbal epithelium, with the expression of CK13 increasing towards the conjunctiva. Nuclei are counterstained with 4',6-diamidino-2-phenylindole (blue); dashed line represents the BM.

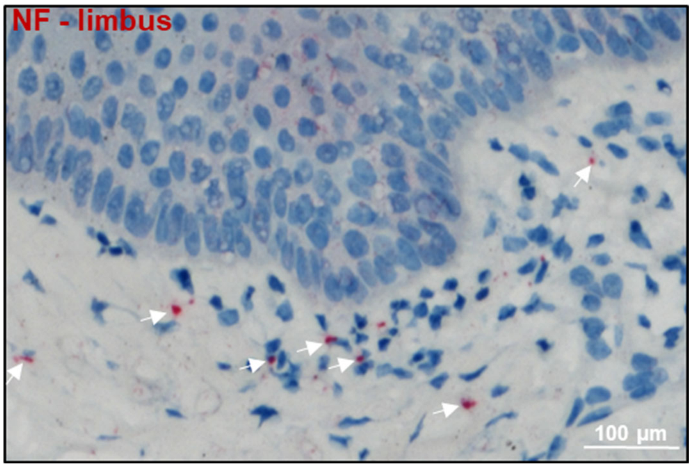

Supplementary Figure S3: Immunohistochemical staining of limbal section showing the neurofilament (NF) expression in the limbal stroma (arrows).

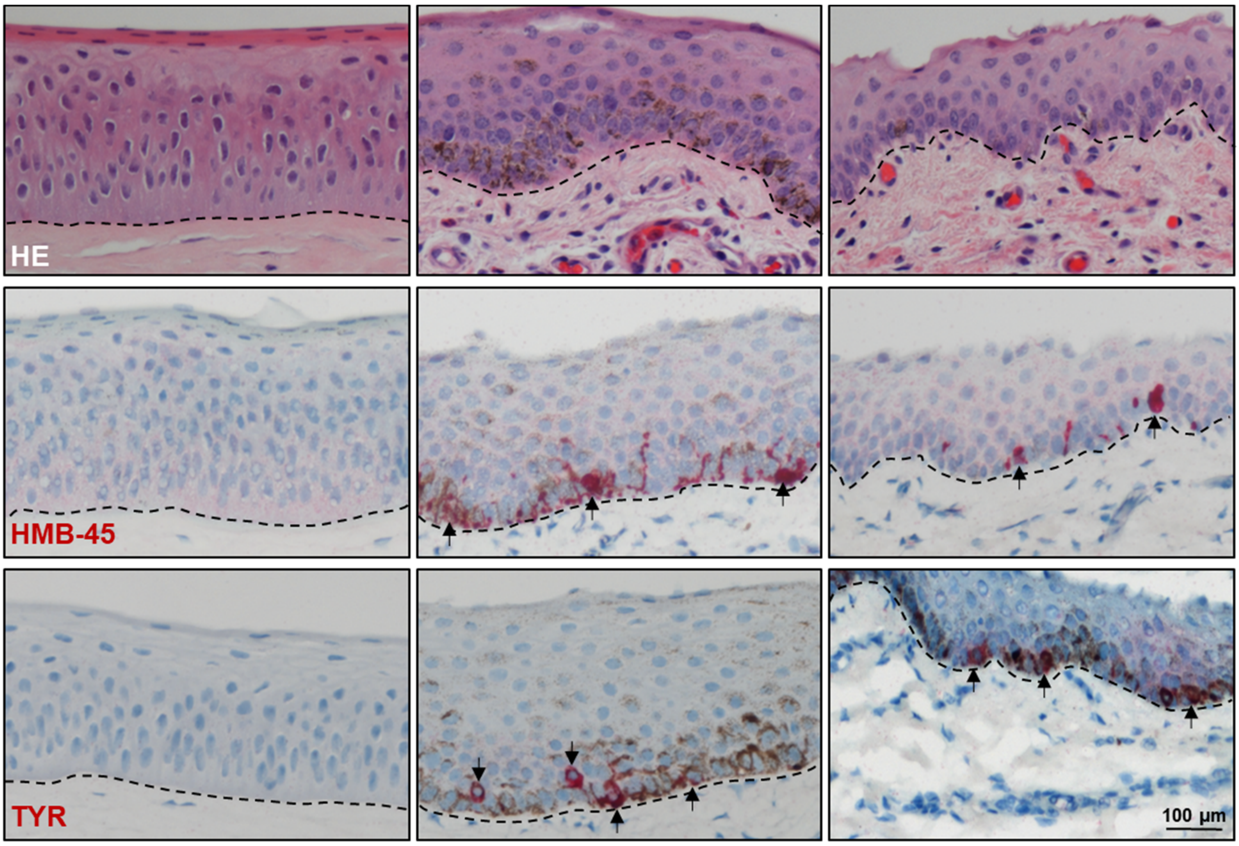

Supplementary Figure S4: Light micrographs of paraffin sections of corneoconjunctival tissue (wild pig) stained with hematoxylin and eosin (H&E); immunohistochemical staining showing the human melanoma black (HMB-45)$^{+}$ and tyrosinase (TYR)$^{+}$ cells in the basal layers of limbal and conjunctival epithelium; dashed line represents basement membrane.
